# Bronchobiliary fistulae as a complication of untreated pulmonary hydatid cyst presenting with bilioptysis: A report of two cases

**DOI:** 10.1002/ccr3.7524

**Published:** 2023-06-13

**Authors:** Parviz Mardani, Hooman Kamran, Fateme Khosravi, Reza Shahriarirad, Pardis Shahabinejad, Bita Geramizadeh, Neda Soleimani, Armin Amirian

**Affiliations:** ^1^ Thoracic and Vascular Surgery Research Center Shiraz University of Medical Science Shiraz Iran; ^2^ Department of Surgery Shiraz University of Medical Sciences Shiraz Iran; ^3^ Student Research Committee Shiraz University of Medical Sciences Shiraz Iran; ^4^ Department of Pathology, School of Medicine Shiraz University of Medical Sciences Shiraz Iran; ^5^ Shiraz Transplant Research Center (STRC) Shiraz University of Medical Sciences Shiraz Iran

**Keywords:** Bronchobiliary, case report, echinococcosis, fistula, pulmonary, surgery

## Abstract

We reported two patients, a 45‐year‐old lady and a 48‐year‐old man, known cases of untreated liver and lung hydatid cysts complicated with bronchobiliary fistulae. Surgery was performed, and bronchobiliary fistulae were diagnosed intraoperatively. Lobectomy was done on the lobe, which was chronically infected. Symptoms resolved after surgery in both cases. Green‐colored sputum in a patient with a history of echinococcosis should raise the physician's attention to the probability of a connection between the bronchial tree and the biliary tract. Surgery in advanced cases is a suitable therapeutic option.

## Graphical abstract

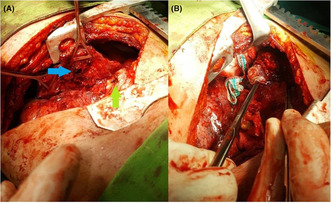

## 1 INTRODUCTION

Cystic echinococcosis (CE) is a universal endemic larval infection that mainly involves the lung and liver^1^, ^2^ but can occur in various organs.^3^ Bronchobiliary fistulae (BBF) is a highly uncommon complication of untreated hydatid cyst, and patients mainly present with bilioptysis.^4^ Clinical manifestation of bilioptysis can attain the diagnosis of BBF, and therapeutic options may include surgical or safer non‐surgical modalities.^5^ We presented two patients with lung and liver hydatid cysts with BBF. In the current report, we share our experience treating this rare entity.

## 2 CASE PRESENTATION

### 2.1 Case 1

A 45‐year‐old lady was admitted to our hospital due to dyspnea, fever, productive cough, and green sputum (bilioptysis). The patient was a known case of lung and liver hydatid cysts. She had a past medical history of pleural effusion due to a rupture of the hydatid cyst 2 years before admission. A preliminary diagnosis of pneumonia was assigned, and a chest tube was inserted due to consolidation and pleural effusion. Recurrence of hydatid cyst was also suspected, and surgery was advised, but the patient did not consent and was discharged after consolidation in her chest was relieved. She received antibiotic therapy for her pneumonia and albendazole to treat the hydatid cyst. Two years later, due to recurrent pneumonia attacks and bilioptysis, she was admitted to our hospital for decortication of the lung hydatid cyst.

**FIGURE 1:**
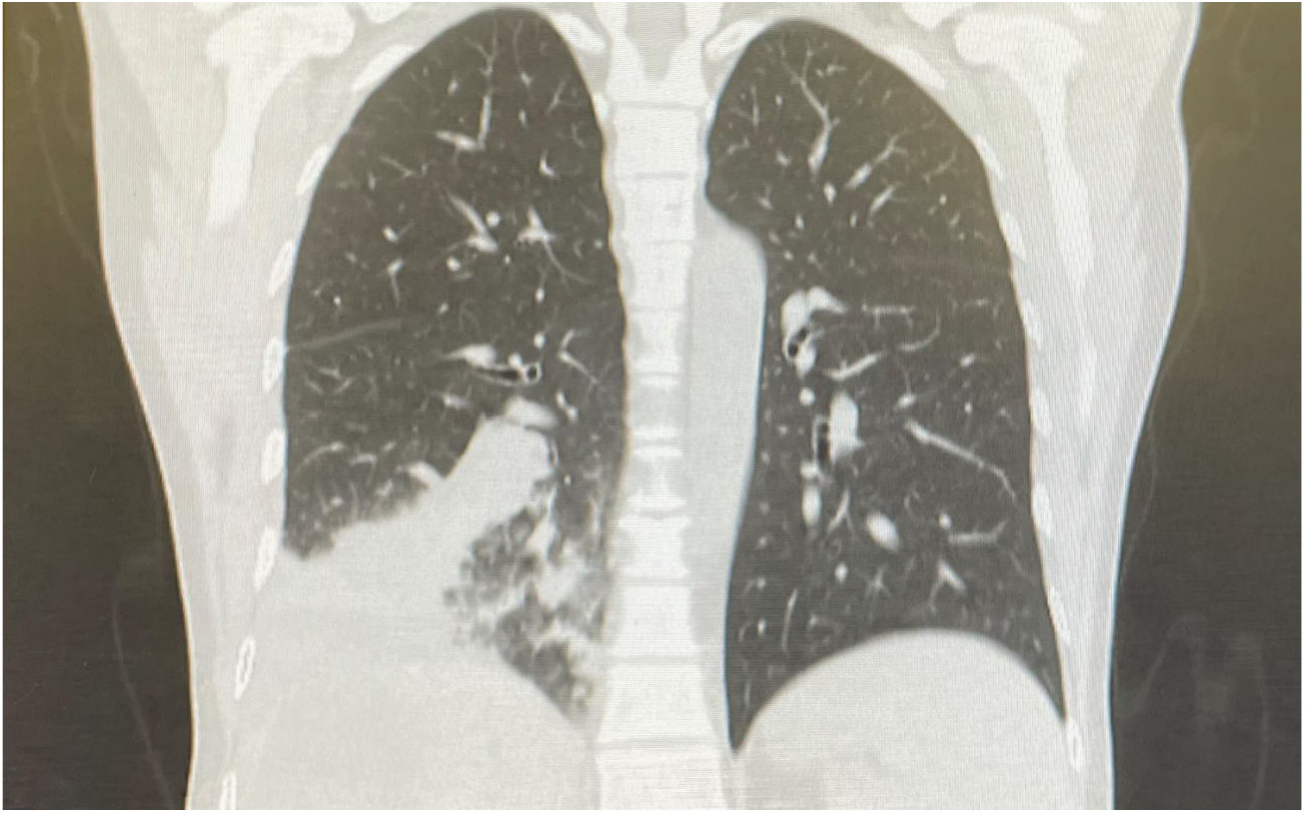
Coronal view of a preoperative chest computed tomography scan of our 45‐year‐old case with bronchobiliary fistula.

We obtained abdominopelvic and chest ultrasonography. Liver containing hypoechoic structure, measuring about 50 × 30 mm in the right lobe of the liver with peripheral calcifications, suggestive of hydatid cyst was reported. Also, chest ultrasonography showed no pleural effusion on the left side and minimal to mild peripheral effusion on the right side. A preoperative chest computed tomography (CT) scan was also provided (Figure 1).

The patient was scheduled for the operation. The operation was performed under general anesthesia in the lateral decubitus position. Due to severe adhesion of the lung, at first, pneumolysis was performed. During pneumolysis of the lower lobe, adhesion to the diaphragm was detected. After meticulous dissection of the lower lobe from the diaphragm, we detected a fistula between the liver and lower lobe of the lung, presumably assumed to be the cause of bilioptysis. Then, via the fistula tract, the hydatid cyst of the liver was removed. Complete irrigation with copious normal saline was done. Also, right lower lobectomy was performed. The diaphragm was rechecked for any fistula or bile leak, in which the fluid was clear without any sign of bile leakage. The diaphragm was subsequently repaired, and two chest tubes were inserted.

**FIGURE 2:**
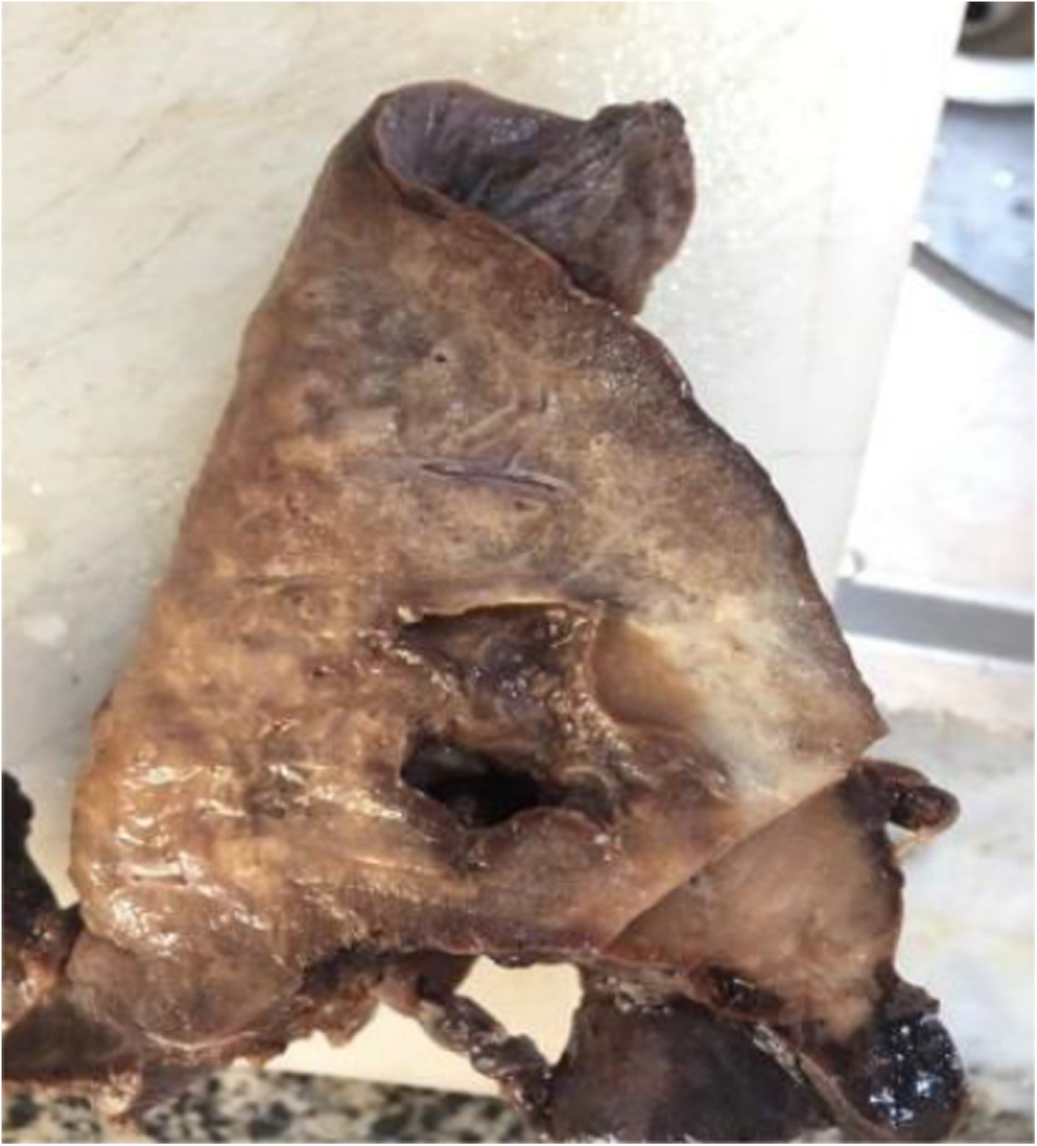
Gross pathology of lower lobe of the lung infected with hydatic cyst and bronchobiliary fistula.

**FIGURE 3:**
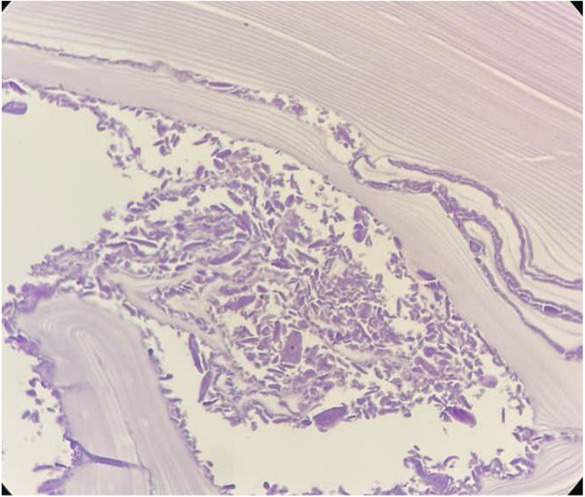
Sections from bronchobiliar fistula show lamellar membrane of hydatid cyst (H&E) (x40).

Obtained tissue samples were sent for histopathological evaluation, in which macroscopic examination showed the lower lobe of the lung with a size of 16 × 14 × 7 cm. The pleural surface was rough and brown in color. A creamy bulging area with a size of 3.5 × 2 × 1 cm was present on the surface. Serial cut sections showed a cystic space with the greatest diameter of 2.5 cm near the base of the lung. Cut sections of the bulging area showed cystic space filled with homogenous creamy material. In other parts of the parenchyma, red color with patchy yellowish discoloration was seen. These findings were in favor of hydatid cyst (Figure 2). Sections from the bronchobiliar fistula showed a lamellar membrane of hydatid cyst (Figure 3).

**FIGURE 4:**
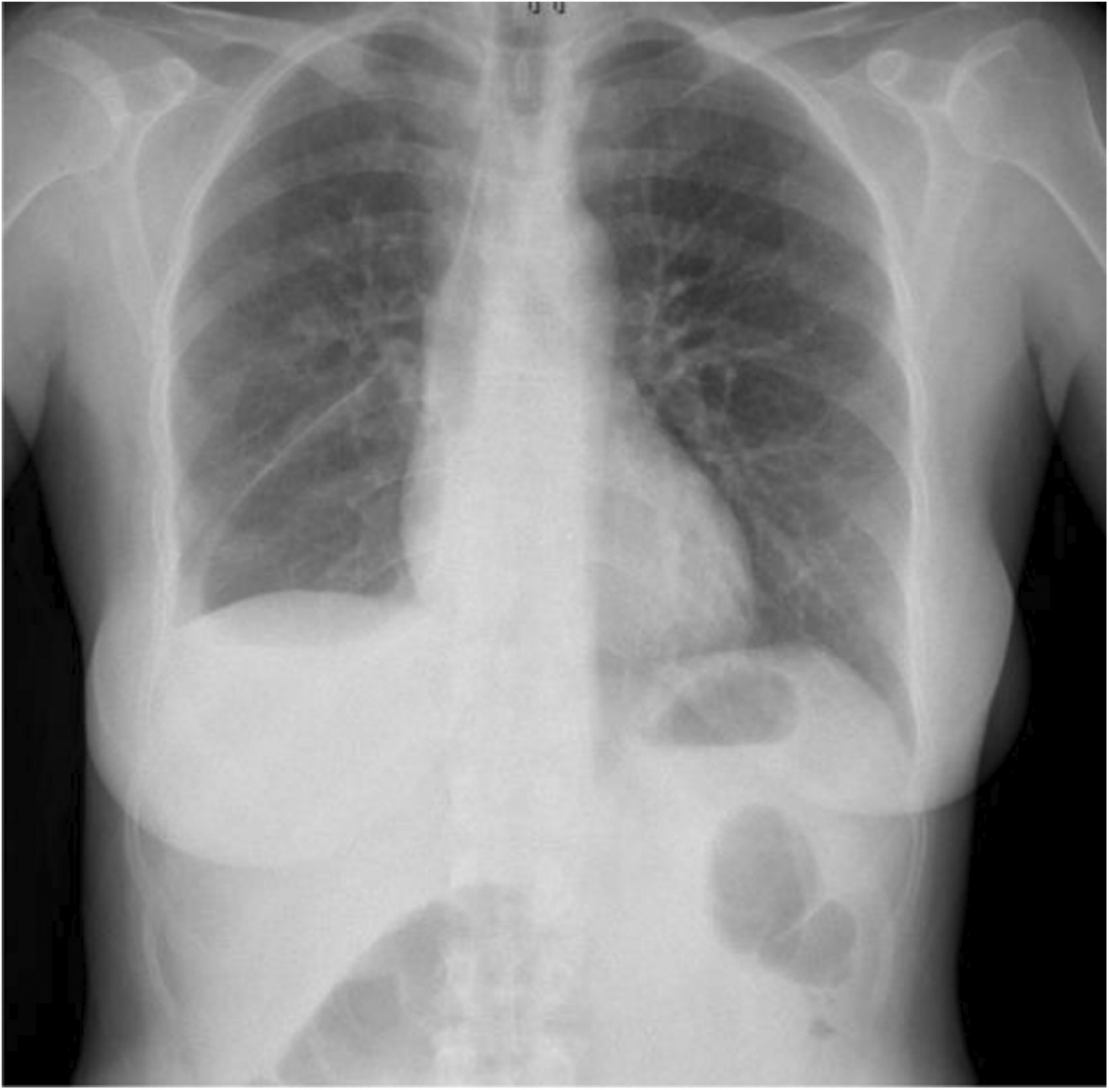
Chest X‐ray of our 45‐year‐old case 5 months following the operation (Case 1).

The patient was discharged in good condition and is on follow‐up every 2 months at a surgical referral clinic. According to chest X‐rays and abdominal ultrasound, no local or systemic recurrence of the cysts has been detected over 9 months (Figure 4).

### 2.2 Case 2

A 48‐year‐old man known case of liver hydatid cyst and previous right lung middle lobe infection was referred to our center with the complaint of abdominal pain and chest discomfort. He reported a history of endoscopic retrograde cholangiopancreatography (ERCP) a year before admission due to a hydatid cyst of the liver. In physical examination, he had stable vital signs.

Previous to his admission at our hospital, a spiral CT had been taken in another center, which showed a large hydatid cyst with exophytic growth arising from the utmost superior part of the right hepatic lobe and extending to the subdiaphragmatic location associated with suspected fistula tract into the thoracic cavity, which indicated a complicated hydatid cyst with hepatopulmonary fistula formation. The suspected fistula was measured at about 20 mm anterior–posterior, 13 mm in transverse, and 30 mm in craniocaudal extension.

**FIGURE 5:**
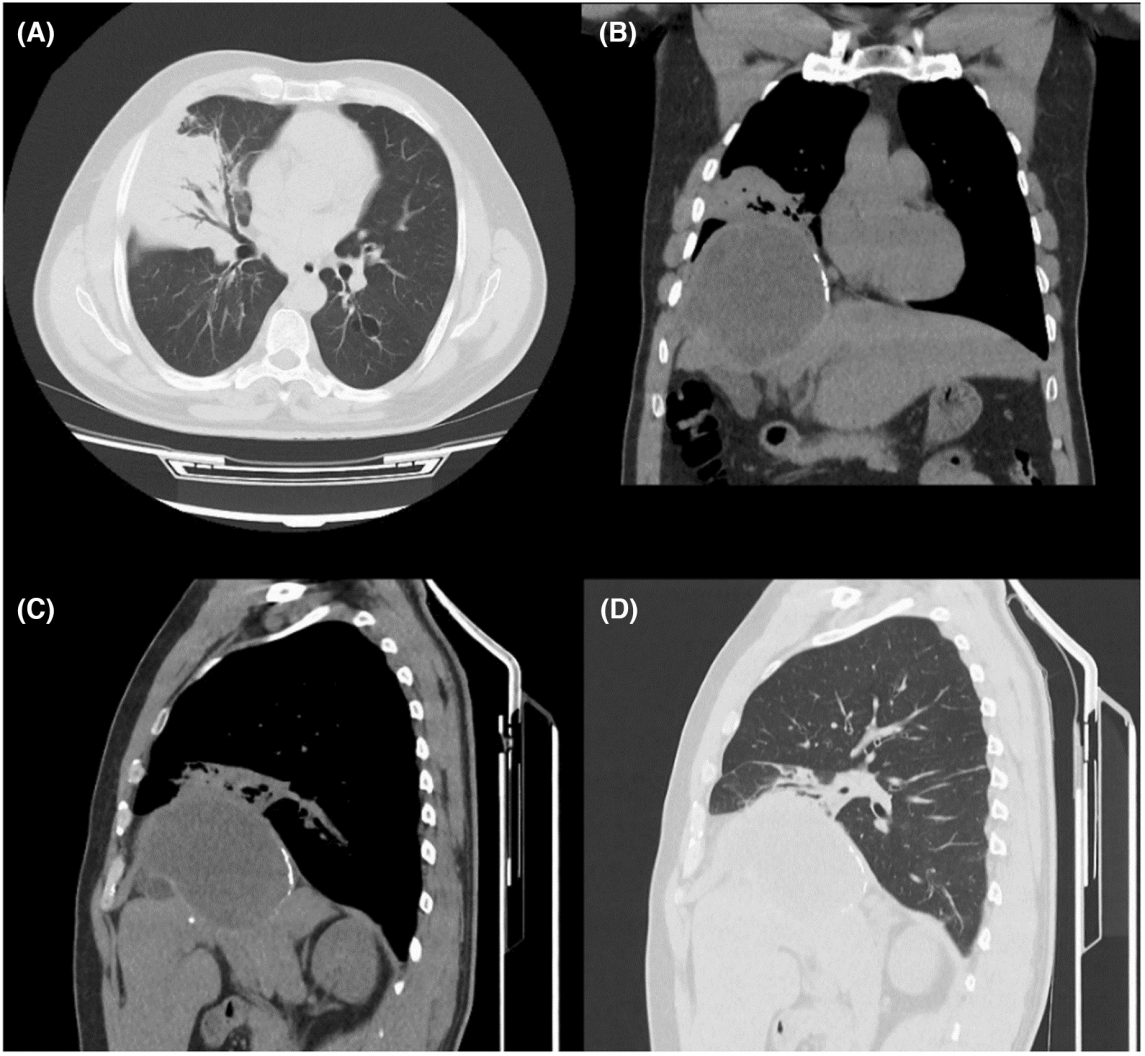
Preoperative chest computed tomography scan of a 48‐year‐old man with bronchobiliary fistula; (A) axial, (B) coronal, and (C), (D) sagittal views.

**FIGURE 6:**
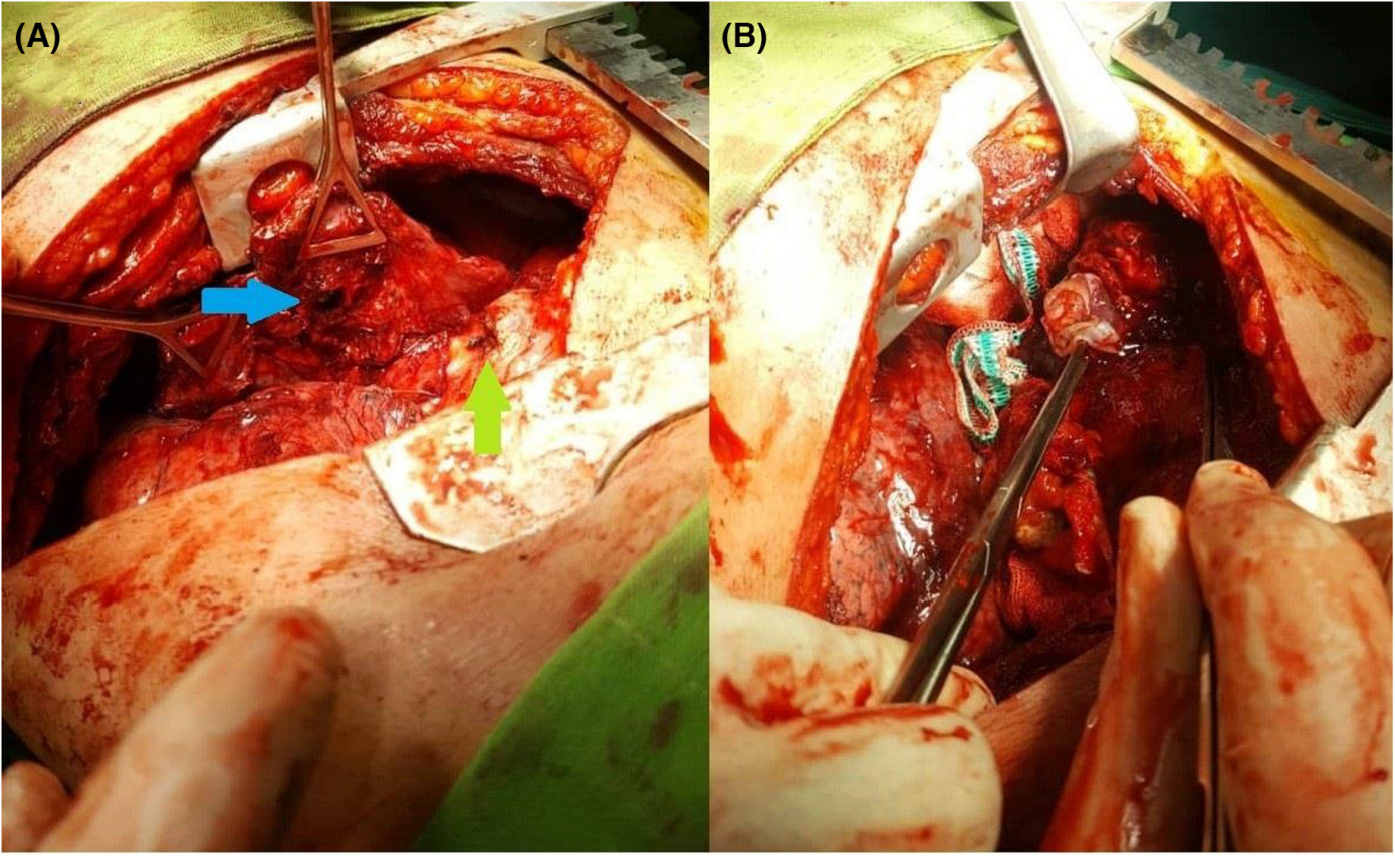
Intraoperative images showing the bronchobiliary fistula; Blue arrow indicator of middle lobe fistula; green arrow indicator of diaphragmatic hole.

**FIGURE 7:**
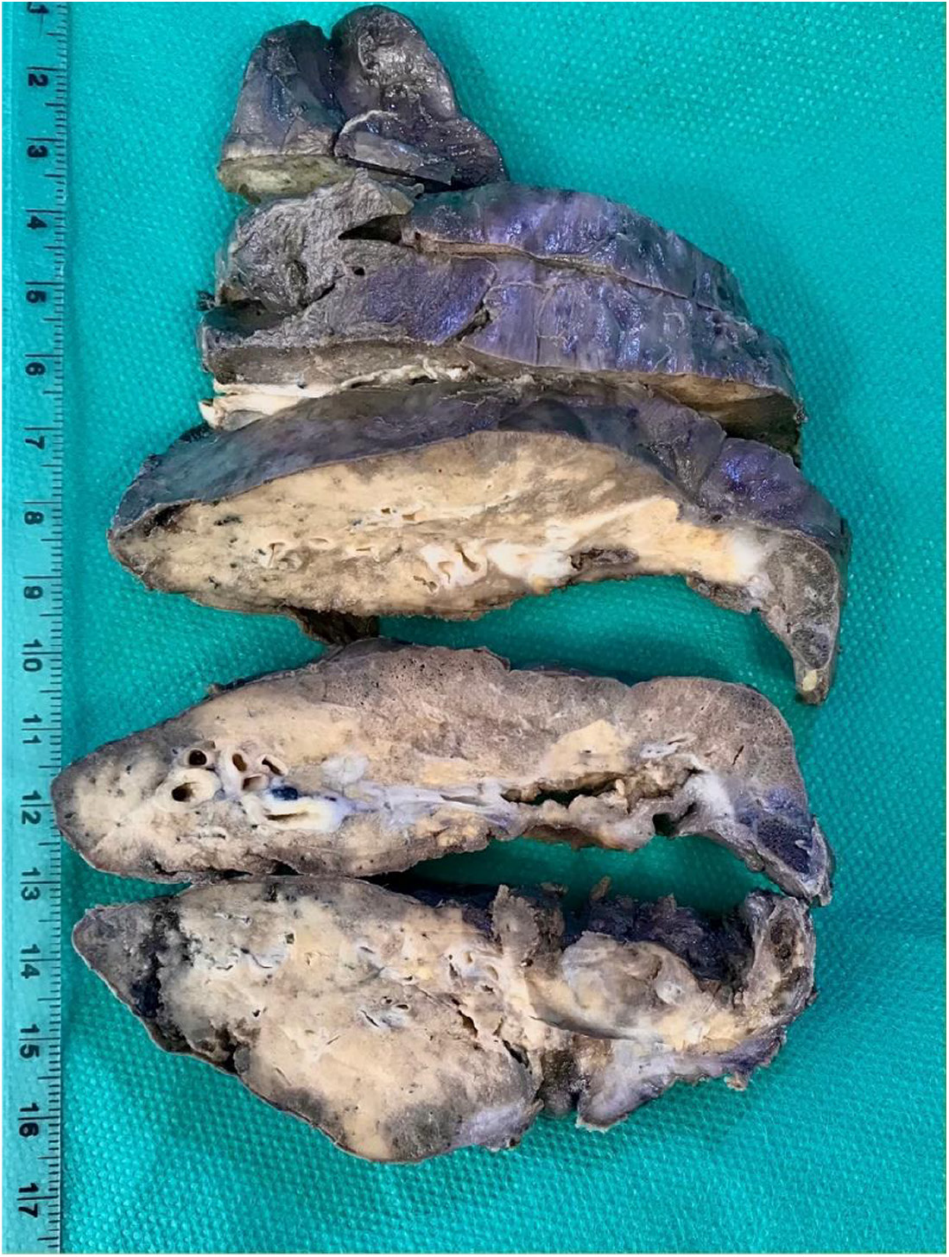
Gross image showing the bronchobiliary fistula in the middle lobe of the lung.

We took another CT scan for the patient (Figure 5) and scheduled the patient for operation. Under general anesthesia, surgery was performed in the lateral decubitus position. At first, open pneumolysis was done due to severe lung adhesion. During pneumolysis of the lower lobe, severe adhesion to the diaphragm was detected. Also, large budging of the diaphragm due to the large hydatid cyst of the liver was observed. In exploration, a fistula tract between the liver and the middle lobe of the lung was seen. The hydatid cyst of the liver was entirely removed via the fistula, and the biliary fistula was closed after complete irrigation. Besides, middle lobectomy and mushroom drain insertion into the liver hydatid cyst via the abdominal wall was performed. The diaphragm was subsequently repaired. The operation ended with the insertion of two chest tubes (Figure 6). Also, Figure 7 shows the gross pattern of the bronchobiliary fistula in the middle lobe of the lung.

**FIGURE 8:**
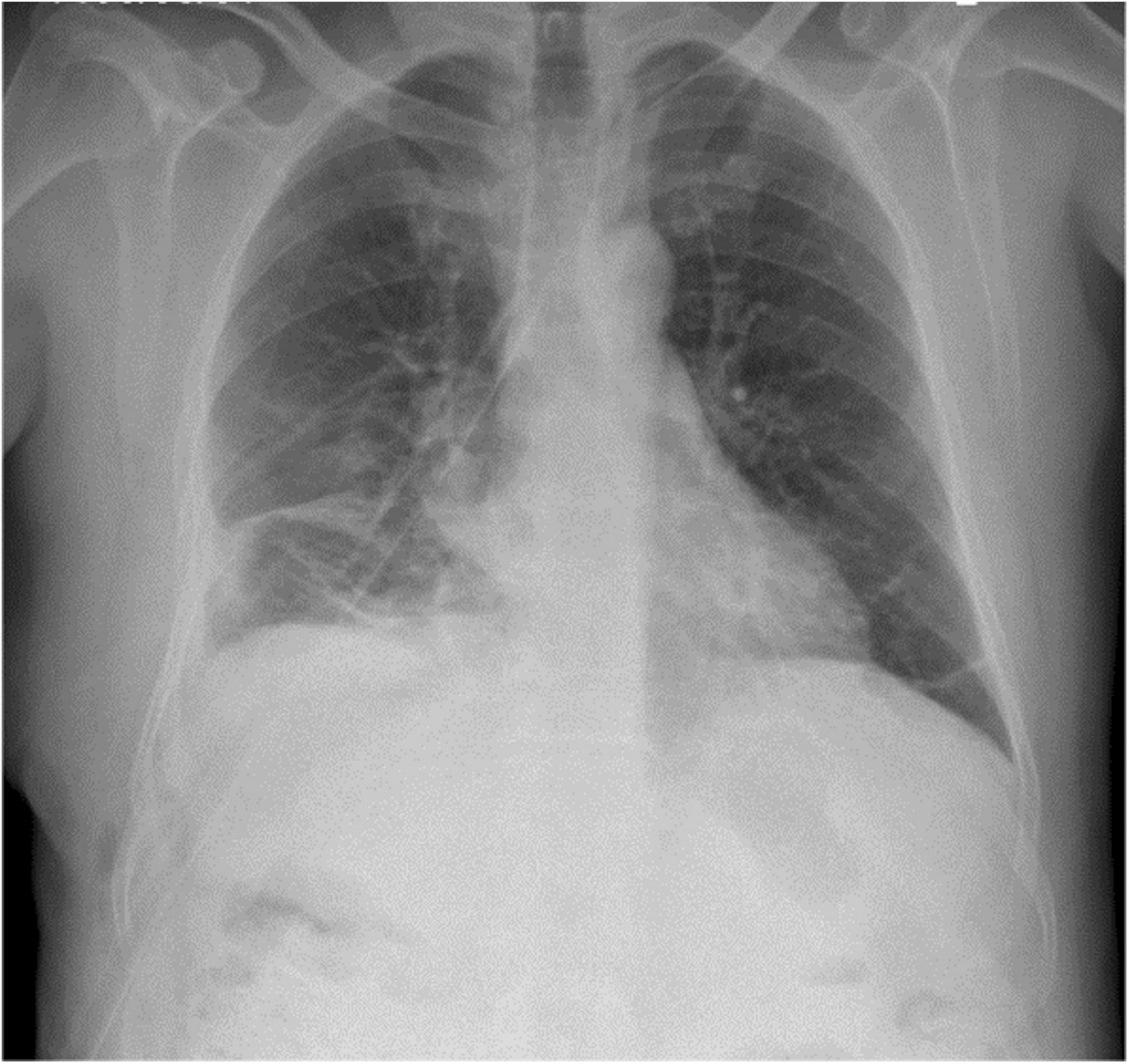
Chest X‐ray of our 48‐year‐old patient a week after the surgery (Case 2).

A chest X‐ray was taken postoperatively (Figure 8), and the patient was discharged in good condition 8 days after the surgery. He is currently under our follow‐up for a month from the surgery, in which he is in relatively good health with no post‐operative complications.

## 3 DISCUSSION

BBF is an extremely rare condition defined as the abnormal interconnection between the right bronchial tree and the biliary tract.^6^ This condition was first diagnosed in 1850 in a patient with hydatid disease.^7^ This condition is either congenital or acquired, developed secondarily to untreated hydatid cyst, hepatic abscess, liver neoplasm, trauma, and surgery.^4^ Our patients were known cases of hydatid cyst who developed with respiratory symptoms. Besides, one of our cases reported bilioptysis. BBF was detected during thoracic surgery while relieving pulmonary adhesions. However, reports suggest that bilioptysis is pathognomonic of BBF,^8^ and diagnosis could be made based on the clinical manifestation of bilioptysis or analysis of sputum.^6^

Diagnosing BBF is challenging, and the only specific sign may be bilioptysis, which can be more or less abundant. The diagnosis should be based on clusters of clinical arguments, paraclinical evidence, and radiological evaluations.^9^ Most of the time, patients with BBF develop accompanying symptoms, like abdominal pain, jaundice, chronic cough, pleuritic chest pain, and fever.^4^, ^10^ BBF can be misdiagnosed with pneumonia at first due to nonspecific respiratory symptoms. Nevertheless, assessment of bilirubin levels in sputum and pleural fluid requires a high level of suspicion. Regarding diagnostic measures, a chest X‐ray could indicate lung collapse or pleural effusion. CT scans can reveal pulmonary, hepatic, or subphrenic collections and parenchymal damage. In these cases, imaging modalities, like ERCP, hepatobiliary scintigraphy (HIDA scan), and magnetic resonance cholangiography (MRCP), could be helpful.^11^

Surgical and non‐surgical interventions are valid measures to treat patients with BBF diagnosis. It is worth mentioning that noninvasive approaches like ERCP, MRCP, and percutaneous transhepatic cholangiography (PTCD) are preferred over traditional surgical approaches in non‐complicated patients with less severe fistulas.^4^, ^5^ Roy J Mukkada and colleagues used endoscopy combined with cyanoacrylate glue and microcoils to treat a stent‐induced BBF.^12^ Tatjana N Adzić‐Vukičević et al. applied percutaneous drainage and antibiotic therapy for echinococcosis induced BBF.^13^ Nan Zhang and colleagues used an endobronchial approach in combination with freeze human fibrinogen.^10^ Rabiou et al.^9^ proposed that the goal of hydatid BBF treatment should be to dry up the fistula, along with treating the cause and repairing the associated abdominothoracic lesion. They mentioned that even in the absence of bilioptysis, surgical treatment is always indicated, in which, in cases of dilation of the bile duct along with obstruction, an initial endoscopic sphincterotomy is indicated to restore biliary flow. Surgery can then be retained following a noticeable decrease in bilioptysis. Furthermore, if general anesthesia is contradicted, endoscopy management can be applied. Therefore, each case of BBF and its therapeutic measures should be evaluated on the patient's condition and symptoms.

In our case, BBF was not suspected before the operation and was detected during thoracotomy. Also, since the patient had repeated pneumonia, fever, and cough, thoracotomy was chosen for the patient. Surgical intervention was the first choice for our patients. It provided us with great access to relieve several pleural and diaphragmatic adhesions, complete hepatic cyst removal, fistula excision, diaphragmatic closure, and lobectomy. This procedure has been used several times in patients with BBF and the management of hydatid cysts^6^, ^14^, ^15^, ^16^, ^17^; surgery is required in advanced cases, like our patients and failed conservative treatments.^5^

Our center is based among the locations considered endemic for cystic echinococcosis. The disease should be adequately managed before presenting any further complications, such as superinfections or, in our case, fistula.^18^, ^19^, ^20^, ^21^ Although BBF in many patients is not considered fatal, intraoperative and post‐operative deaths have been reported.^5^, ^22^ Our patients' outcomes were excellent, and symptoms resolved after surgery.

## 4 CONCLUSION

In summary, green‐colored sputum in a patient with a history of echinococcosis should raise the physician's attention to the probability of a connection between the bronchial tree and the biliary tract. Surgery in advanced cases, like our patients, is a suitable therapeutic option with a satisfactory prognosis.

## AUTHOR CONTRIBUTIONS

**Parviz Mardani:** Conceptualization; investigation; supervision. **Hooman Kamran:** Data curation; resources; writing – original draft. **Fateme Khosravi:** Writing – original draft. **Reza Shahriarirad:** Data curation; investigation; project administration; supervision; writing – original draft; writing – review and editing. **Pardis Shahabinejad:** Writing – original draft. **Bita Geramizadeh:** Data curation; investigation; supervision; validation. **Neda Soleimani:** Data curation; investigation; validation. **Armin Amirian:** Investigation; supervision.

## FUNDING INFORMATION

No financial support was received for this case report.

## CONFLICT OF INTEREST STATEMENT

The authors declare that they have no competing interests.

## ETHICS STATEMENT

The present study was approved by the Medical Ethics Committee of Shiraz University of Medical Sciences. The purpose of this study was completely explained to the patients and was assured that their information would be kept confidential by the researchers.

## CONSENT

Written informed consent was obtained from the patient to publish this report in accordance with the journal's patient consent policy.

## Data Availability

All data regarding this case report has been reported in the manuscript. Please contact the corresponding author in case of requiring any further information.
